# 8-Hydroxyeicosapentaenoic Acid Decreases Plasma and Hepatic Triglycerides via Activation of Peroxisome Proliferator-Activated Receptor Alpha in High-Fat Diet-Induced Obese Mice

**DOI:** 10.1155/2016/7498508

**Published:** 2016-04-28

**Authors:** Hidetoshi Yamada, Sayaka Kikuchi, Mayuka Hakozaki, Kaori Motodate, Nozomi Nagahora, Masamichi Hirose

**Affiliations:** ^1^Iwate Biotechnology Research Center, 22-174-4 Narita, Kitakami, Iwate 024-0003, Japan; ^2^Department of Molecular and Cellular Pharmacology, Iwate Medical University School of Pharmaceutical Sciences, Shiwa, Iwate 028-3694, Japan

## Abstract

PPARs regulate the expression of genes involved in lipid homeostasis. PPARs serve as molecular sensors of fatty acids, and their activation can act against obesity and metabolic syndromes. 8-Hydroxyeicosapentaenoic acid (8-HEPE) acts as a PPAR ligand and has higher activity than EPA. However, to date, the PPAR ligand activity of 8-HEPE has only been demonstrated* in vitro*. Here, we investigated its ligand activity* in vivo* by examining the effect of 8-HEPE treatment on high fat diet-induced obesity in mice. After the 4-week treatment period, the levels of plasma and hepatic triglycerides in the 8-HEPE-fed mice were significantly lower than those in the HFD-fed mice. The expression of genes regulated by PPAR*α* was significantly increased in 8-HEPE-fed mice compared to those that received only HFD. Additionally, the level of hepatic palmitic acid in 8-HEPE-fed mice was significantly lower than in HFD-fed mice. These results suggested that intake of 8-HEPE induced PPAR*α* activation and increased catabolism of lipids in the liver. We found no significant differences between EPA-fed mice and HFD-fed mice. We demonstrated that 8-HEPE has a larger positive effect on metabolic syndrome than EPA and that 8-HEPE acts by inducing PPAR*α* activation in the liver.

## 1. Background

An imbalance between energy intake and expenditure can result in the accumulation of excess triglycerides in adipose tissues. Long-term continuation of this imbalance can result in chronic obesity, which is associated with hyperlipidemia, fatty liver, and adipocyte hypertrophy. Hyperlipidemia is a risk factor for atherosclerosis [1] and is also a predisposing factor for cardiovascular diseases. Fatty liver is another risk factor for cardiovascular disease and is thought to have a negative influence on the regulation of insulin signaling [2]. Adipocyte hypertrophy contributes to dysfunction in the adipose tissues and is associated with insulin resistance and increased risk of developing diabetes [3]. As a chronic high level of triglycerides is a common factor for hyperlipidemia, fatty liver, and adipocyte hypertrophy, any increase in triglyceride expenditure should potentially alleviate the risk of cardiovascular diseases and diabetes.

PPARs are members of a nuclear receptor superfamily and play critical roles in the regulation of storage and catabolism of lipids [4]. They contribute to these regulation processes by activating gene expression in a ligand-dependent manner, which involves recognition of and binding to peroxisome proliferator response elements (PPREs) that are composed of TGACCT-related direct repeats separated by one nucleotide [5, 6]. PPARs form heterodimers on PPREs via the retinoid-X receptor, the receptor for 9-cis-retinoic acid [7, 8]. Three types of PPAR have been identified, namely, *α*, *γ*, and *δ*. PPAR*α* is expressed at high levels in the liver where it promotes fatty acid oxidation, ketogenesis, lipid transport, and gluconeogenesis [9, 10]. PPAR*α* responds to the concentration of fatty acids in the liver and enhances fatty acid breakdown by upregulating genes encoding *β*-oxidation enzymes [11–13]. Phenoxyalkylcarboxylic acid derivatives (fibrates) have been used to treat hypertriglyceridemia through activation of PPAR*α* [14–16]. Activation of PPAR by fibrates results in a substantially reduced level of serum triglycerides [16]. However, these drugs have the adverse side effects of hepatic toxicity, myopathy, and cholelithiasis. Thus, other PPAR activators are being investigated to determine whether they show fewer adverse effects than fibrates.

The identification of unsaturated fatty acids as PPAR ligands provided firm evidence that the direct interaction of nuclear receptors with these fatty acids is required for some PPAR-dependent transcription activity [7, 11, 17–20]. Unsaturated fatty acids can bind to all three types of PPAR, with PPAR*α* exhibiting the highest affinity for concentrations equivalent to circulating blood levels [11, 21]. In contrast, the long-chain fatty acid erucic acid (C22:1) is a weak ligand that appears to have more affinity for PPAR*δ* [22]. Overall, saturated fatty acids are poor PPAR ligands compared to unsaturated fatty acids [7, 11, 19]. Hydroxyeicosapentaenoic acids (HEPEs) are unsaturated fatty acids and are the oxylipin products of the lipoxygenase pathway. In a previous study, we showed that dried pacific krill is a source of HEPEs (5-HEPE, 8-HEPE, 9-HEPE, 12-HEPE, and 18-HEPE) and that 8-HEPE has high ligand activity for PPARs [23]. 8-HEPE increases the levels of expression of genes regulated by PPARs in Fao (rat hepatoma cell line), 3T3-F442A (mouse preadipocyte cell line), and C2C12 (mouse myoblast cell line) cells. Furthermore, 8-HEPE enhances adipogenesis and glucose uptake. By contrast, at the same concentrations, eicosapentaenoic acid (EPA) shows only a weak effect, indicating that 8-HEPE is a more potent inducer of physiological effects.

8-HEPE has a greater affinity for PPAR activation than EPA* in vitro*; it is possible that 8-HEPE might be of value in the treatment of obesity and metabolic syndrome. Pacific krill contain about 20 mg of 8-HEPE per 100 g [23] and therefore could potentially be used as a food supplement. However, no animal experiments on the* in vivo* effectiveness of 8-HEPE have been reported. Here, we treated high-fat diet-induced obese mice with 8-HEPE to investigate its antiobesity effects.

## 2. Material and Methods

### 2.1. 8-HEPE and EPA Purification from Pacific Krill

8-HEPE and EPA were purified as described previously [23]. Dried krill (Kawashu, Iwate, Japan) were powdered and then extracted with methanol. The extract was subjected to column chromatography using Diaion HP-20 (Mitsubishi Chemical, Tokyo, Japan). The 8-HEPE- and EPA-containing fraction was eluted with methanol from the HP-20. 8-HEPE and EPA were separated on an InertSustain ODS-3 column (20.0 mm dia. × 250 mm; GL Science Inc.).

### 2.2. Animal Experiments

Four-week-old, male C57BL/6J mice (Charles River Laboratories, Tokyo, Japan) were housed singly in a temperature-controlled environment (23 ± 1°C) with a 12 h light/dark cycle. The animal experiments reported here were approved by the institutional animal care and use committee at Iwate Biotechnology Research Center (IBRC-ARC-2014-01). The low-fat diet AIN-93G (protein 13.9% calorie, fat 9.7% calorie, and carbohydrate 77.0% calorie) (total 377 kcal/100 g diet) and the high-fat diet HFD-60 (protein 18.2% calorie, fat 62.2% calorie, and carbohydrate 19.6% calorie) (total 506 kcal/100 g diet) were purchased from Charles River Laboratories. The animals were maintained on HFD for seven weeks. Then, they were randomly assigned to six groups, with 9 mice in each group: HFD; HFD with EPA (10 mg/kg); HFD with 8-HEPE (10 mg/kg); low-fat diet (LFD); LFD with EPA (10 mg/kg); and LFD with 8-HEPE (10 mg/kg). EPA and 8-HEPE were added to diet without exchange of other dietary components. The animals were maintained on these diets for 4 weeks prior to all analyses. Food intake was measured twice a week, and body weight was measured weekly. Blood samples were collected using heparin sodium (Wako) coated tubes, and plasma was separated from the blood by centrifugation at 14000 g for 10 min.

### 2.3. Measurement of Triglycerides, Glucose, Cholesterol, Transaminase, Leptin, and Adiponectin

Hepatic lipids were extracted from mouse livers as described by Folch et al. [32]. In brief, the liver (~50 mg) was homogenized in 700 *μ*L of 0.1 M acetic acid solution-methanol (2 : 5, v/v). Chloroform (500 *μ*L) was added to the mixture and, after 10 min, 250 *μ*L of 0.1 M acetic acid was added. The mixture was centrifuged at 2400 g for 10 min at room temperature. The chloroform layer was allowed to evaporate and the pellet was suspended in 200 *μ*L of isopropanol. Triglycerides in blood plasma and liver extracts were measured using the Triglyceride E-Test (Wako). Total cholesterol, glucose, and transaminase in plasma were measured using the Total Cholesterol E-Test (Wako), Glucose C2-Test (Wako), and Transaminase C2-Test (Wako), respectively. Plasma leptin was measured using a mouse leptin measurement kit (Morinaga Institute of Biological Science, Inc., Kanagawa, Japan). Plasma adiponectin was measured using an Adiponectin ELISA Kit (Otsuka Pharmaceutical Co., Ltd., Tokushima, Japan). All kits were used according to the manufacturers' recommendations.

### 2.4. Measurement of Palmitic Acid and Stearic Acid in Liver

A 100 mg piece of the liver was homogenized in 1 mL of cold methanol/water (4 : 1, v/v). After sonication, the sample was placed on ice for 20 minutes and then deproteinized by centrifugation at 21,000 g for 10 min. The supernatant (800 *μ*L) was freeze-dried and dissolved in 100 *μ*L of methanol/water (4 : 1, v/v) before analysis. Palmitic acid and stearic acid were separated on an InertSustain ODS-3 column (2.0 mm dia. × 250 mm; GL Science Inc.) with gradient elution (acetonitrile/water/formic acid, 30/70/0.1 to 90/10/0.1 in 30 min) at a flow rate of 0.2 mL/min. The compounds were identified and quantified by LC-TOFMS using Agilent Mass Hunter Workstation Software. Analytes were quantified with internal standard methods and 5-point calibration curves. Assay variability was assessed by analyzing sample replicate.

### 2.5. Quantitative Real-Time PCR

Total RNAs were extracted with an RNeasy Lipid Tissue Kit (QIAGEN, Tokyo, Japan) and used to synthesize cDNAs using a PrimeScript RT Reagent Kit (Takara, Shiga, Japan); all kits were used according to the manufacturers' recommendations. Quantitative real-time PCR was performed with the gene specific primers listed in Supplemental Table 1 in Supplementary Material available online at http://dx.doi.org/10.1155/2016/7498508 and Fast SYBR Green Master Mix (Applied Biosystems, Foster City, CA, USA).

### 2.6. Histology

Gonadal fat depots were fixed in 10% formalin (Wako) for 3 hours and embedded in paraffin (Wako). Paraffin embedded samples were sectioned at 6 *μ*m thickness with a microtome (Leica, Tokyo, Japan). Sections were subjected to standard hematoxylin and eosin staining. Adipocyte area was measured with ImageJ software (http://rsbweb.nih.gov/ij/).

### 2.7. Statistical Analysis

Statistically significant differences between the experimental groups were identified using one-way ANOVA and Tukey's post hoc tests. Data are shown as means ± SD.

## 3. Results

### 3.1. Food Intake and Weight Gain

We did not find any significant differences among the groups of mice for food intake per day or body weight (see Figures S1 and S2 and Table 1). Based on food intake, the mice ingested 23.98 ± 1.99 *μ*g of EPA or 23.67 ± 1.69 *μ*g of 8-HEPE per day, respectively.

### 3.2. Plasma Triglyceride Levels Are Suppressed in Mice Fed 8-HEPE

Plasma triglyceride levels in mice fed HFD with 8-HEPE were significantly lower than in the HFD group (Table 2). There was no significant difference in plasma triglyceride levels between mice fed HFD with EPA and mice fed HFD or among the mice fed LFD, LFD with EPA, and LFD with 8-HEPE (Table 2). 8-HEPE consumption did not affect plasma glucose or total cholesterol levels.

### 3.3. 8-HEPE Reduces Triglyceride Accumulation in Liver

Next, we examined the effect of 8-HEPE on the liver. Triglyceride levels in mice fed HFD with 8-HEPE were significantly lower than the HFD group (Table 2). As fatty liver is the most common hepatic disorder in Western countries [24], we examined liver function in the three groups of mice by measuring aspartate aminotransferase (AST) and alanine aminotransferase (ALT) activities in the plasma. The levels of activity of these enzymes in the plasma are used as a marker to indicate hepatic disorders. ALT activity in mice fed 8-HEPE was lower than the HFD group (Table 2).

**Figure 1:**
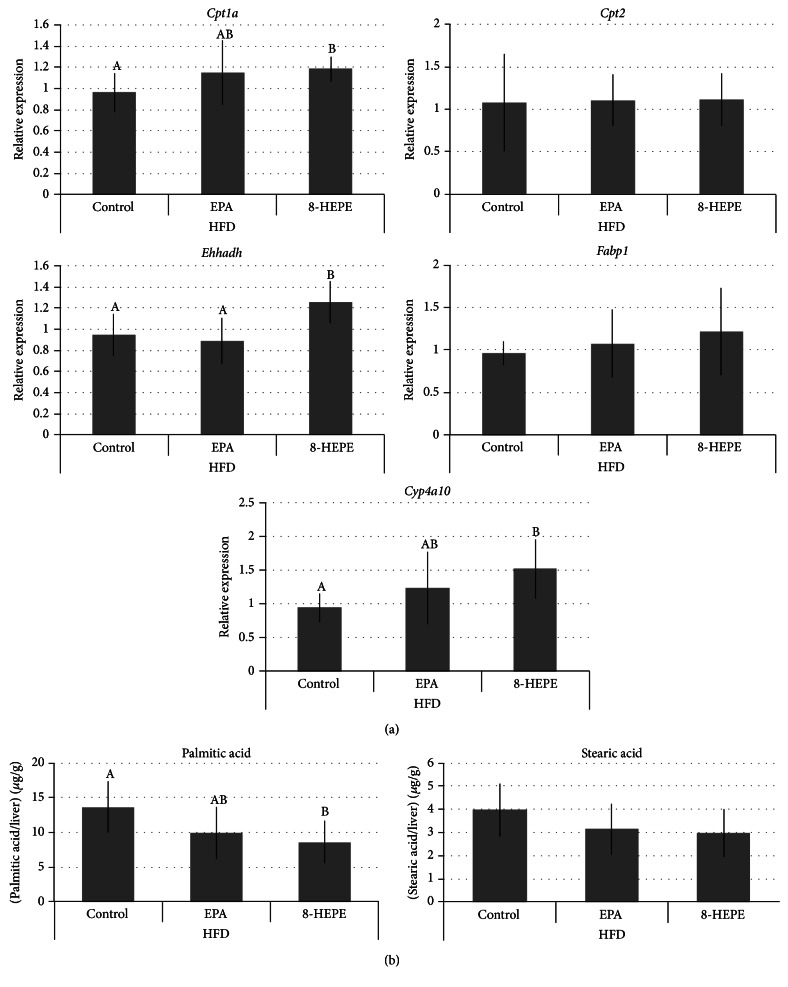
Changes in hepatic gene expression (a) and in concentrations of long-chain saturated fatty acids (b) in male C57BL/6J-DIO mice fed HFD, HFD with EPA, or HFD with 8-HEPE for 4 wk. Gene expression levels were measured by real-time PCR and normalized against expression of* Actb*. Values are means ± SDs, *n* = 9. Labeled means without a common letter differ; *P* < 0.05. HFD: high-fat diet; HEPE: hydroxyeicosapentaenoic acid; EPA: eicosapentaenoic acid.

In our previous study, we showed that 8-HEPE acts as a PPAR*α* ligand in the rat hepatoma cell line FaO [23]. Moreover, Wy14,643, a PPAR*α* agonist, induces expression of* Cpt1a*,* Cpt2*,* Ehhadh*,* Fabp1*, and* Cyp4a* in the liver [25, 26]. Here, we used real-time PCR to determine whether 8-HEPE consumption affects gene expression levels in the mouse liver. We found that the level of* Cpt1a*,* Ehhadh,* and* Cyp4a10* expression in mice fed HFD with 8-HEPE was higher than in mice fed HFD (Figure 1(a)). However, there was no significant difference in hepatic gene expression levels between mice fed LFD and mice fed LFD with 8-HEPE (Figure S3). We also investigated expression of sterol regulatory element binding transcription factor 1 (*Srebf1*) and fatty acids synthase (*Fasn*) as it has been reported that EPA can suppress hepatic lipogenesis and steatosis by reducing transcription of* Srebf1* [27]. However, we could not detect any change in* Srebf1* or* Fasn* expression in the mice fed 8-HEPE (Figure S4). The mice fed HFD or HFD with EPA showed no significant differences for liver weight, liver triglyceride levels, plasma AST or ALT activities, and gene expression levels (Table 2 and Figure 1). In the mice fed HFD with 8-HEPE, the palmitic acid level was significantly lower than in the HFD group (Figure 1(b)). These results indicate that 8-HEPE activates PPAR*α* and increases fatty acid oxidation in liver.

### 3.4. 8-HEPE Reduces Adipocyte Hypertrophy

**Figure 2:**
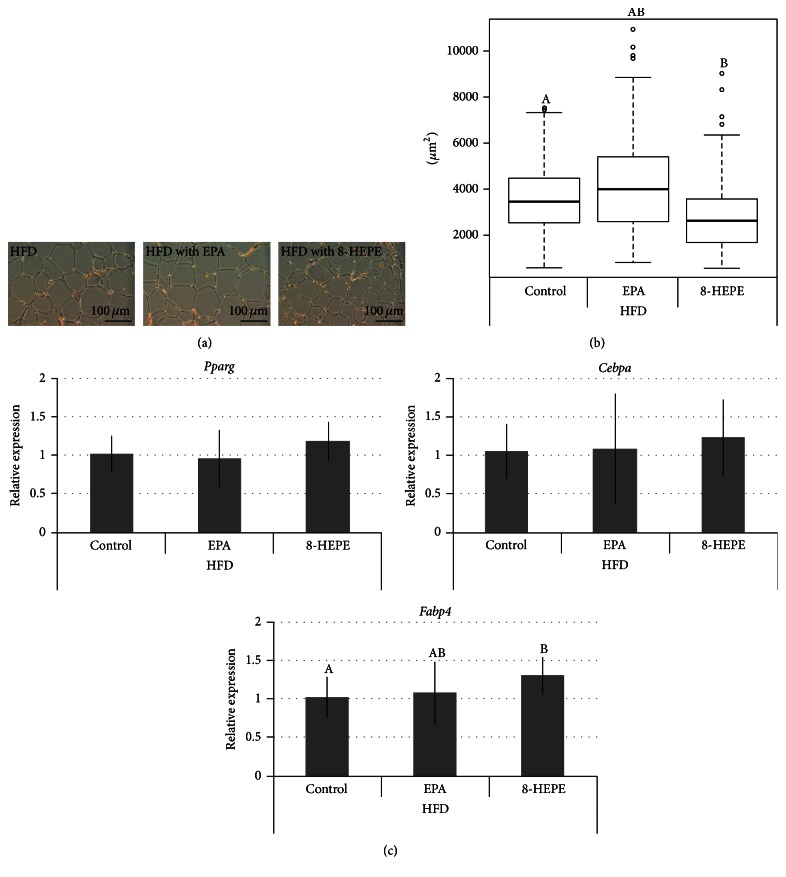
Hematoxylin and eosin staining of gonadal WAT (a), quantification of adipocyte cross sections (b), and expression of* Pparg*,* Cebpa,* and* Fabp4* in gonadal WAT (c) in male C57BL/6J-DIO mice fed HFD, HFD with EPA, or HFD with 8-HEPE for 4 wk. Data were collected from H&E-stained sections from five mice; two fields of view were analyzed per mouse, and 10–15 cells per field were analyzed using ImageJ software. Gene expression levels were measured by real-time PCR and normalized against expression of* Actb*. Values are means ± SDs, *n* = 9. Labeled means without a common letter differ; *P* < 0.05. HFD: high-fat diet; HEPE: hydroxyeicosapentaenoic acid; EPA: eicosapentaenoic acid.

Obesity is associated with adipocyte hypertrophy and functional disorder of the adipose tissue [28]. To determine whether 8-HEPE ameliorated these effects, we compared gonadal white adipose tissue (gonadal WAT) in the three groups of mice. We found that relative gonadal WAT was slightly reduced in the mice fed HFD with 8-HEPE compared to the HFD group (Table 1). Moreover, the gonadal adipocyte cells were smaller in the mice fed 8-HEPE compared to those fed HFD (Figures 2(a) and 2(b)). As adipose tissue regulates energy homeostasis and insulin sensitivity via the secretion of leptin and adiponectin [29], we measured the concentrations of adiponectin and leptin in the plasmas of the mice. No significant differences were present between mice fed HFD and HFD with 8-HEPE (Table S4). Our previous study showed that 8-HEPE can activate PPAR*γ* in mice preadipocyte cells [23]. In line with this finding, mice fed 8-HEPE showed increased expression of* Fabp4* in gonadal WAT (Figure 2(c)); however, they did not show any difference in expression of* Pparg* and* Cebpa*. The activation of PPAR*δ* induces* Angptl4* expression in muscle [23] and* Lpin2* and* St3gal5* in the liver [30]. To estimate PPAR*δ* activation here, we measured* Angptl4*,* Lpin2,* and* St3gal5* expression; however,* Angptl4*,* Lpin2,* and* St3gal5* did not show any increase in expression level in mice fed 8-HEPE (Figures S4 and S5).

## 4. Discussion

Our analyses here demonstrate that 8-HEPE intake reduces the levels of triglycerides in the blood and the liver and also lessens adipocyte hypertrophy in mice. Mice fed HFD with 8-HEPE consumed 23.67 ± 1.69 *μ*g/day of 8-HEPE and had an average body weight of 32.3 g. Thus, the relative 8-HEPE consumption was approximately 0.73 mg/kg/day. Compared to the control HFD diet, 8-HEPE was associated with a decrease in plasma triglyceride levels (Cohen's *d* = 0.64), in liver triglycerides (Cohen's *d* = 1.01), and in gonadal WAT (Cohen's *d* = 0.49) (Tables 1 and 2). However, supplementation of the diet with 8-HEPE did not alter daily food intake or plasma glucose and cholesterol levels (Tables 2, S1, and S2). Compared to the LFD diet, 8-HEPE did not affect plasma triglycerides, hepatic triglycerides, or hepatic gene expression. These results indicate that the major effect of 8-HEPE was to reduce triglyceride levels in mice fed a high-fat diet intake. The consequent reduction in plasma ALT activity in the 8-HEPE mice (Table 2) suggests that this supplement might aid in the prevention of fatty liver induced hepatic disorders in the mice. Previous studies have shown that EPA administration is effective in decreasing plasma triglyceride levels in experimental animals and humans [31]. However, in the present study, there were no significant differences in plasma triglyceride levels between mice fed HFD and HFD with EPA (Table 2). The contrasting outcomes of EPA on plasma triglycerides may be due to differences in the dose of EPA.

Both Forman et al. and our group have reported that 8-HEPE can act as a PPAR ligand [11, 23]. However, this is the first study to report on the* in vivo* effects of 8-HEPE on PPARs. As detailed above, the expression of genes regulated by PPAR*α*, such as* Cpt1a*,* Ehhadh,* and* Cyp4a10*, was increased in the livers of mice fed 8-HEPE (Figure 1(a)). These results indicate that 8-HEPE taken orally can activate PPAR*α* and cause a decrease in triglyceride levels in the liver. Moreover, 8-HEPE was detected in the plasma of mice fed 8-HEPE (Table S3). We also found that* Fabp4* expression was increased in the gonadal WAT of mice fed 8-HEPE (Figure 2(c)). However, plasma concentrations of adiponectin and leptin were not altered in these mice (Table S4). In view of our results, the daily intake of 8-HEPE used here appeared to be too low to activate PPAR*γ*. PPAR*δ* activation was not observed either (Figures S4 and S5). Overall, our results indicate that a 0.73 mg/kg/day intake of 8-HEPE in mice is sufficient to activate PPAR*α* in the liver, but that activation of PPAR*γ* and PPAR*δ* may require higher levels of 8-HEPE intake.

## 5. Conclusions

This study is the first demonstration that supplementation of the diet with 8-HEPE can have a beneficial effect on various characteristics of obesity through the activation of PPAR*α*. When we compared mice fed 8-HEPE to those fed unmodified HFD, statistically significant changes were observed in plasma and liver triglyceride levels and the amount of gonadal WAT. Statistically significant effects were not observed, if the mice were fed a diet supplemented with EPA. Thus, 8-HEPE induced a higher level of lipid catabolism in liver than EPA. Notably, 8-HEPE can easily be obtained from krill for use as a food supplement. Our results here indicate that use of 8-HEPE as a supplement may offer protective effects against hyperlipidemia, fatty liver, and adipose tissue dysfunction in patients with chronic obesity.

## Supplementary Material

Experimental design (Figure S1, S2), compositions of the diets (Table S1), primers for real-time PCR (Table S2), concentration of plasma 8-HEPE in mice (Table S3), concentration of plasma adiponectin and leptin in mice (Table S4), hepatic gene expression changes (Figure S3, S4) and Muscle *Angptl4* gene expression change (Figure S5) are shown in supplemental material.

## Figures and Tables

**Table 1:** Body weights, liver weights, the relative amount of gonadal WAT per g body weight in male C57BL/6J-DIO mice fed HFD, HFD with EPA, HFD with 8-HEPE, LFD, LFD with EPA, or LFD with 8-HEPE for 4 wk. Values are means ± SDs, *n* = 9. Labeled means without a common letter differ; *P* < 0.05. HFD: high-fat diet; LFD: low-fat diet; HEPE: hydroxyeicosapentaenoic acid; EPA: eicosapentaenoic acid.

	Body weight (g)	Liver weight (g)	Gonadal WAT per g body weight (%)
HFD	35.96 ± 2.19^a^	1.28 ± 0.13^a^	5.94 ± 0.61^a^
HFD with EPA	35.92 ± 2.95^a^	1.26 ± 0.13^a^	5.85 ± 1.13^ab^
HFD with 8-HEPE	35.09 ± 1.27^a^	1.29 ± 0.10^a^	5.74 ± 0.39^b^
LFD	26.02 ± 1.17^b^	0.96 ± 0.22^b^	2.16 ± 0.40^c^
LFD with EPA	25.42 ± 3.64^b^	1.02 ± 0.14^b^	2.71 ± 0.66^c^
LFD with 8-HEPE	26.10 ± 1.46^b^	1.00 ± 0.12^b^	2.36 ± 0.48^c^

**Table 2:** Plasma triglycerides, glucose, total cholesterol, AST, ALT, and hepatic triglyceride in male C57BL/6J-DIO mice fed HFD, HFD with EPA, HFD with 8-HEPE, LFD, LFD with EPA, or LFD with 8-HEPE for 4 wk. Values are means ± SDs, *n* = 9. Labeled means without a common letter differ; *P* < 0.05. HFD: high-fat diet; LFD: low-fat diet; HEPE: hydroxyeicosapentaenoic acid; EPA: eicosapentaenoic acid.

	Plasma					Hepatic
	Triglyceride (mg/dL)
HFD	91.36 ± 22.14^a^	335.23 ± 59.78	172.04 ± 16.35^a^	30.85 ± 3.59	10.88 ± 2.15^a^	50.18 ± 10.87^a^
HFD with EPA	82.10 ± 23.53^ab^	327.58 ± 37.75	168.27 ± 21.07^a^	32.45 ± 6.87	10.86 ± 3.53^ab^	44.31 ± 13.79^ab^
HFD with 8-HEPE	78.12 ± 18.44^b^	327.05 ± 47.14	166.13 ± 14.38^a^	30.35 ± 3.41	9.30 ± 1.39^b^	40.53 ± 7.65^b^
LFD	74.76 ± 25.10^ab^	308.01 ± 28.80	103.07 ± 9.12^b^	31.76 ± 4.24	5.93 ± 1.13^c^	29.27 ± 5.32^c^
LFD with EPA	70.99 ± 44.21^ab^	279.73 ± 25.72	109.13 ± 17.21^b^	32.33 ± 6.19	6.73 ± 1.39^c^	28.47 ± 4.92^c^
LFD with 8-HEPE	68.14 ± 23.10^b^	319.66 ± 52.42	107.02 ± 11.98^b^	35.71 ± 7.15	7.72 ± 1.43^c^	30.85 ± 8.52^c^

## Abbreviations

HEPE: Hydroxyeicosapentaenoic acid
PPARs: Peroxisome proliferator-activated receptors
EPA: Eicosapentaenoic acid
Cyp4a10: Cytochrome P450 CYP4A enzymes
Fabp: Fatty acid-binding protein
Cpt: Carnitine palmitoyltransferase
Ehhadh: Enoyl-CoA hydratase/3-hydroxyacyl CoA.
