# Supplementary material for: 8-Hydroxyeicosapentaenoic Acid Decreases Plasma and Hepatic Triglycerides via Activation of Peroxisome Proliferator-Activated Receptor Alpha in High-Fat Diet-Induced Obese Mice
Source: J Lipids. 2016 Apr 28;2016:7498508. doi: 10.1155/2016/7498508 (PMC4864551; doi:10.1155/2016/7498508)
Supplement: Supplementary file 1 — Experimental design (Figure S1, S2), compositions of the diets (Table S1), primers for real-time PCR (Table S2), concentration of plasma 8-HEPE in mice (Table S3), concentration of plasma adiponectin and leptin in mice (Table S4), hepatic gene expression changes (Figure S3, S4) and Muscle Angptl4 gene expression change (Figure S5) are shown in supplemental material. [file 7498508.f1.pdf]

Supplemental Material

Measurement of 8-HEPE in plasma

Blood plasma was mixed with acetonitrile in 1M acetic acid (ratio, 1:3). The samples were centrifuged and the supernatants collected. The supernatants were concentrated on a MonoSpin C18 column (GL Science Inc.). The extraction efficiency is 92 ± 4.3 %. 8-HEPE and EPA were separated on an InertSustain ODS-3 column (2.0 mm dia. × 250 mm; GL Science Inc.) with gradient elution (acetonitrile/water/formic acid, 30/70/0.1 to 90/10/0.1 in 30 min) at a flow rate of 0.2 mL/min. The compounds were identified and quantified by liquid chromatography-time of flight mass spectrometry (LC-TOFMS) (Agilent Technologies) using Agilent Mass Hunter Workstation Software (Agilent Technologies).

Supplemental Table 1

	HFD-60 (High-fat diet)	AIN-93M (Low-fat diet)
	(g / kg diet)	
Casein	200	140
L-cystine	3.6	1.8
Mart-Dextrin	60	-
Cornstarch	-	465.692
Alfarized cornstarch	160	155
Sucrose	55	100
Soybean oil	20	40
Lard	330	-
Cellulose	66.1	50
AIN-93 mineral mixture	35	35
AIN-93 vitamin mixture	10	10
Calcium carbonate	1.8	-
Choline bitartrate	2.5	2.5
Tert-butylhydroquinone	-	0.008

Supplemental Table 1. Compositions of the diets.

Supplemental Table 2

Symbol	Organism	Description	Ref seq DNA ID	Real-time PCR primers
Actb	Mus musculus	beta actin	NM_007393	TGGAATCCTGTGGCATCCATGAAAC
				TAAAACGCAGCTCAGTAACAGTCCG
Fabp1	Mus musculus	Fatty acid binding protein 1, liver	NM_017399	GTGGTCCGCAATGAGTTCAC
				CACCTTCCAGCTTGACGACT
Cyp4a10	Mus musculus	cytochrome P450, family 4, subfamily a, polypeptide 10	NM_010011	TTCCAGCAGTTCCCATCACC
				CTTGCTTCCCCAGAACCATCT
Ehhadh	Mus musculus	Enoyl-Coenzyme A, hydratase/3-hydroxyacyl Coenzyme A dehydrogenase	NM_023737	GGCTAGAGCCCTGCAGTACGC
				CGATGCCTCGGCCCATCGTTC
Cpt1a	Mus musculus	Carnitine palmitoyltransferase 1a, liver	NM_013495	TGGACCCCTCCCTGGGCATG
				ATCGCCACCCAGAGCCCTGT
Cpt2	Mus musculus	Carnitine palmitoyltransferase 2	NM_009949	CAGTGTGGGCGAGCTTCAGC
				TCAGGCAGGGTGACCCCTCT
Fabp4	Mus musculus	Fatty acid binding protein 4, adipocyte	NM_024406	GATGCCTTTGTGGGAACCT
				CTGTCGTCTGCGGTGATTT
Pparg	Mus musculus	Peroxisome proliferator activated receptor gamma	NM_00112733 01	TGCCTTCGCTGATGCACTGCC
				CACGGAGAGGTCCACAGAGCTGA
Cepba	Mus musculus	CCAAT/enhancer binding protein, alpha	NM_007678	CCAACCCCATCCCCAACGGC
				GAAGCGGTCCAGCCCTGCTC
Adipoq	Mus musculus	Adiponectin	NM_009605	GCACTGGCAAGTTCTACTGCAA
				GTAGGTGAAGAGAACGGCCTTGT
Srebf1	Mus musculus	Sterol regulatory element binding transcription factor 1	NM_011480	GGACACAGCGGTTTTGAACG
				CTCAGGAGAGTTGGCACCTG
Fasn	Mus musculus	Fatty acid synthase	NM_007988	ATCTCTCCAAGTTCGACGCC
				GTTCGTTCCTCGGAGTGAGG
Lpin2	Mus musculus	Lipin 2	NM_00116488 5	TGCTTTACCTAGAAGATAACAGTGA
				CCAGCTGGCCCACATAATTC
St3gal5	Mus musculus	ST3 beta-galactoside alpha-2,3-sialyltransfera se 5	NM_00103522 8	CAATGGTACACCCGAACCCA
				AGGAGCCAGACTCCAAATGC

Angptl4	Mus musculus	Angiopoietin-like 4	NM_020581	ACTTGGGACCAAGACCATGAC
				TACAGGTACCAAACCACCAGC

Supplemental Table 2. Primers for real-time PCR

Supplemental Table 3

Mice Groups	8-HEPE
HFD	n.d.
HFD with EPA	n.d.
HFD with 8-HEPE	3.7 ± 1.9

Supplemental Table 3. Concentration (nM) of 8-HEPE in male C57BL/6J-DIO mice fed HFD, HFD with EPA, or HFD with 8-HEPE for 4 wk. Values are means ± SDs, n = 9. Means at a time without a common letter differ, $P < 0.05$. HFD, High fat diet; HEPE, hydroxyeicosapentaenoic acid; EPA, eicosapentaeic acid;

Supplemental Table 4

	Plasma adiponectin (µg / mL)	Plasma leptin (ng / mL)
HFD	26.53 ± 3.09	41.25 ± 10.63
HFD with EPA	27.45 ± 3.61	40.17 ± 14.89
HFD with 8-HEPE	26.51 ± 2.58	35.79 ± 7.07

Supplemental Table 4. Concentrations of plasma adiponectin and leptin in male C57BL/6J-DIO mice fed HFD, HFD with EPA or HFD with 8-HEPE for 4 wk. Values are means ± SDs, n = 9. HFD, high fat diet; HEPE, hydroxyeicosapentaenoic acid; EPA, eicosapentaeic acid;

Supplemental Fig. 1

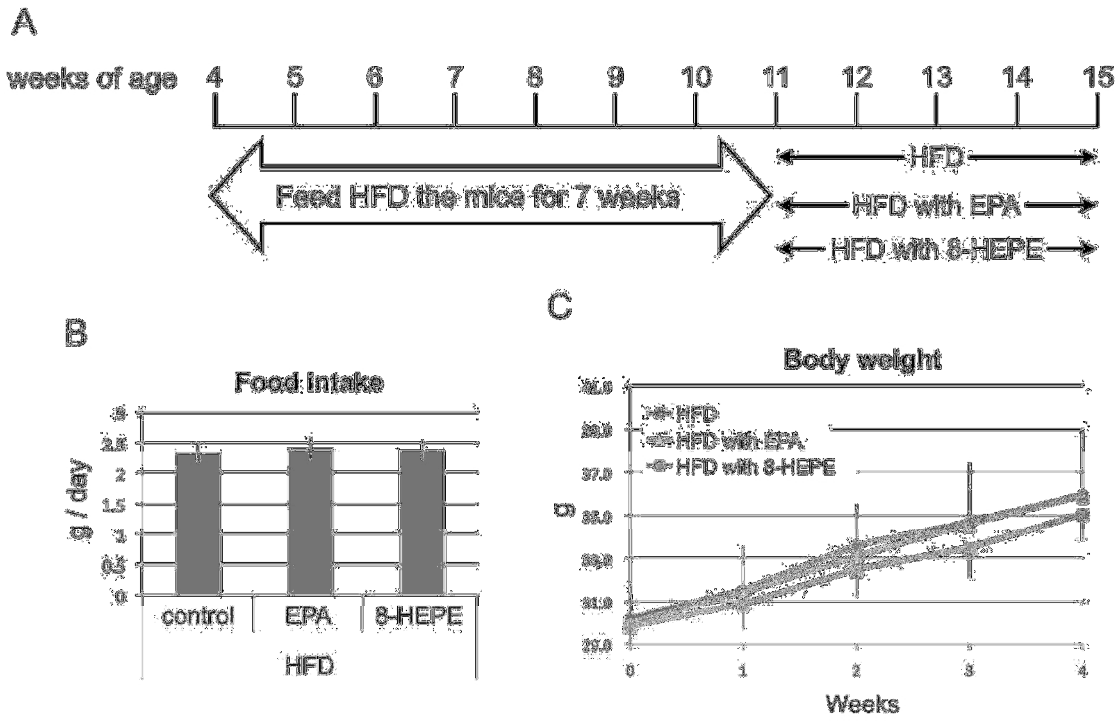

Supplemental Figure 1. Experimental design (A), food intake (B) and body weight gain (C) in male C57BL/6J-DIO mice fed HFD, HFD with EPA, or HFD with 8-HEPE for 4wk. Values are means ± SDs, n = 9. HFD, High fat diet; HEPE, hydroxyeicosapentaenoic acid; EPA, eicosapentaeic acid;

Supplemental Fig. 2

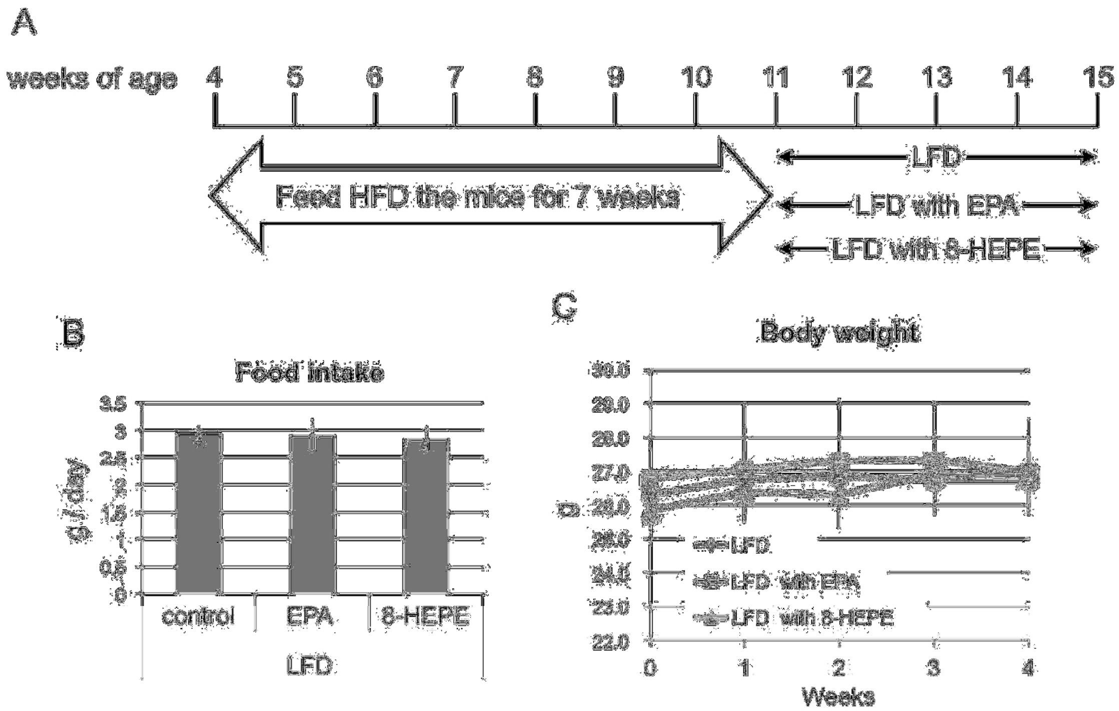

Supplemental Figure 2. Experimental design (A), food intake (B) and body weight gain (C) in male C57BL/6J-DIO mice fed LFD, LFD with EPA, or LFD with 8-HEPE for 4wk. Values are means ± SDs, n = 9. LFD, Low fat diet; HEPE, hydroxyeicosapentaenoic acid; EPA, eicosapentaeic acid;

Supplemental Fig. 3

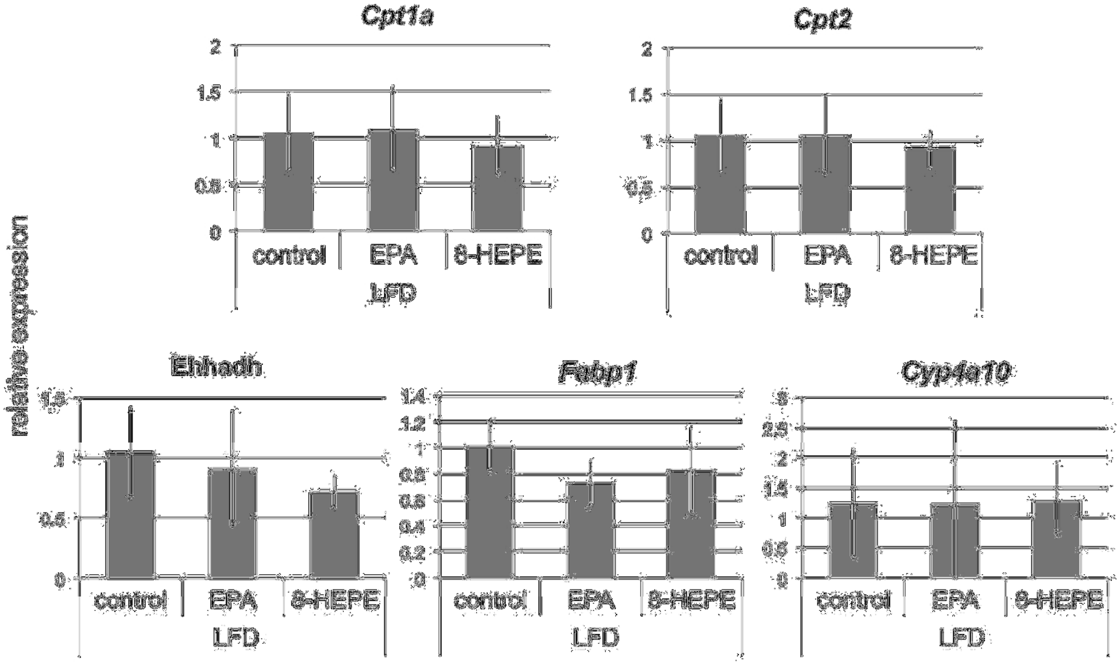

Supplemental Figure 3. Hepatic gene expression changes in male C57BL/6J-DIO mice fed LFD, LFD with EPA, or LFD with 8-HEPE for 4wk. Gene expression levels were measured by real-time PCR and normalized against expression of *Actb.* Values are means ± SDs, n = 9. LFD, Low fat diet; HEPE, hydroxyeicosapentaenoic acid; EPA, eicosapentaeic acid;

Supplemental Fig. 4

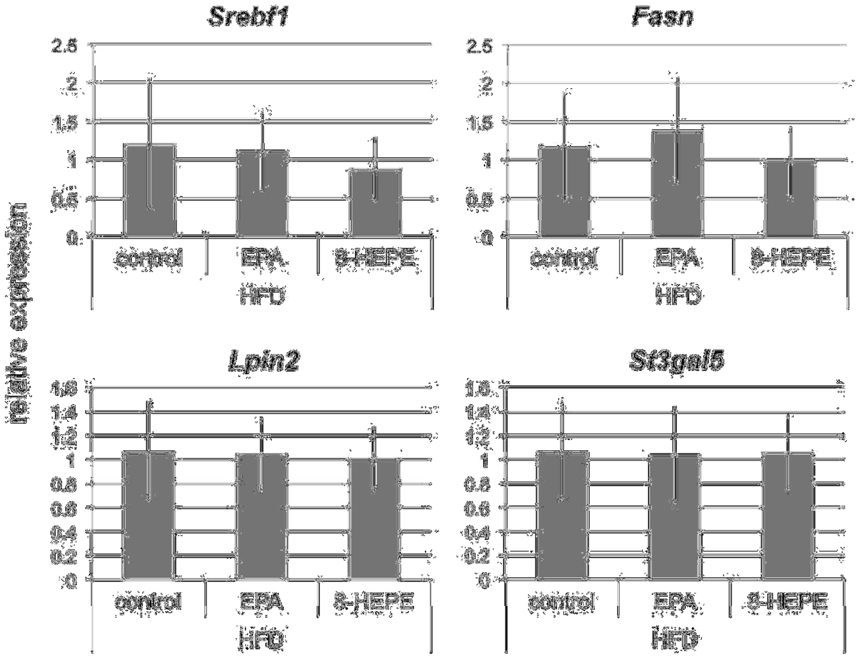

Supplemental Figure 4. Hepatic gene expression changes in male C57BL/6J-DIO mice fed HFD, HFD with EPA, or HFD with 8-HEPE for 4wk. Gene expression levels were measured by real-time PCR and normalized against expression of *Actb.* Values are means ± SDs, n = 10. HFD, High fat diet; HEPE, hydroxyeicosapentaenoic acid; EPA, eicosapentaeic acid;

Supplemental Figure 5

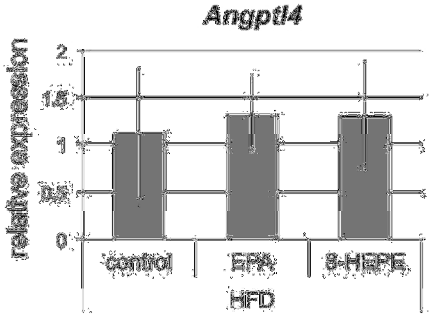

Supplemental Figure 5. Muscle *Angptl4* gene expression change in male C57BL/6J-DIO mice fed HFD, HFD with EPA, or HFD with 8-HEPE for 4wk. Gene expression levels were measured by real-time PCR and normalized against expression of *Actb.* Values are means ± SDs, n = 10. HFD, High fat diet; HEPE, hydroxyeicosapentaenoic acid; EPA, eicosapentaeic acid;
